# Measurement of Cetuximab and Panitumumab-Unbound Serum EGFR Extracellular Domain Using an Assay Based on Slow Off-Rate Modified Aptamer (SOMAmer) Reagents

**DOI:** 10.1371/journal.pone.0071703

**Published:** 2013-08-21

**Authors:** Noh Jin Park, Xiuqiang Wang, Angelica Diaz, Dana M. Goos-Root, Christopher Bock, Jonathan D. Vaught, Weimin Sun, Charles M. Strom

**Affiliations:** 1 Quest Diagnostics Nichols Institute San Juan Capistrano, San Juan Capistrano, California, United States of America; 2 SomaLogic Incorporated, Boulder, Colorado, United States of America; Penn State College of Medicine, United States of America

## Abstract

**Background:**

Response to cetuximab (Erbitux®) and panitumumab (Vectibix®) varies among individuals, and even those who show response ultimately gain drug resistance. One possible etiologic factor is differential interaction between the drug and target. We describe the development of an assay based on Slow Off-rate Modified Aptamer (SOMAmer^™^) reagents that can distinguish drug-bound from unbound epidermal growth factor receptor (EGFR).

**Methods:**

This quantitative assay uses a SOMAmer reagent specific for EGFR extracellular domain (ECD) as a capturing reagent. Captured SOMAmer is quantitated using PCR. Linearity and accuracy (recovery) of the assay were assessed using normal sera and purified EGFR ECD.

**Results:**

This EGFR ECD assay showed linearity between 2.5 and 600 ng/mL. Average recovery was 101%. The assay detected EGFR but showed little cross-reactivity to other ErbB proteins: 0.4% for ErbB2, 6.9% for ErbB3, and 1.3% for ErbB4. Preincubation of normal serum with either cetuximab or panitumumab resulted in a dose-dependent decrease in EGFR ECD levels measured using the SOMAmer assay; preincubation did not affect measurement with an ELISA.

**Conclusions:**

This SOMAmer-based serum EGFR ECD assay accurately and specifically measures EGFR in serum. Detection of significant amounts of drug-unbound EGFR in patients undergoing cetuximab or panitumumab treatment could be an indicator of poor drug response. Further studies are needed to evaluate the utility of the assay as an indicator of drug efficacy or as a guide to dosing.

## Introduction

EGFR, also known as Her-1 and ErbB1, is a well characterized oncogene that codes for a member of the tyrosine kinase ErbB family [1]. It is a 170 kDa glycoprotein located on the surface of epithelial cells. Binding of its ligands, such as epidermal growth factor (EGF), amphiregulin, transforming growth factor- α (TGF-α), betacellulin, epiregulin, heparin-binding EGF-like growth factor (HB-EGF), and epigen, induces EGFR homodimerization as well as heterodimerization with erbB2 (HER-2/neu), erbB3 (HER3), or erbB4 (HER4). Dimerization results in activation of the intracellular kinase domains, tyrosine autophosphorylation, and internalization of the receptor-ligand complex. This signaling cascade regulates multiple biological functions including cell proliferation, differentiation, motility, and apoptosis. Alterations in the structure, expression, and signaling of EGFR may be involved in the development and metastasis of a wide variety of cancers.

The EGFR protein is divided into three domains: a glycosylated extracellular domain (ECD) that binds growth factors; a short transmembrane portion; and an intracellular tyrosine kinase portion responsible for signal transduction. The ECD can be released into the circulation via proteolytic cleavage or alternative splicing [2], [3].

Although tumor tissues have been shown to over-express EGFR protein, cancer patients often show a decrease (40%–60%) in serum EGFR ECD levels compared to normal control subjects [4]. As the cancer stage advances, a higher percentage of serum samples have ECD levels below the normal range. The reason for detecting low levels of circulating ECD among patients whose tumors overexpress EGFR remains unclear. In addition, some forms of cancer are associated with increased levels of circulating EGFR ECD. Due to the inconsistent expression pattern of circulating EGFR ECD in different tumor types, EGFR alone may not be a suitable maker for cancer diagnosis or prognosis. However, it can be used in conjunction with other tumor-specific markers.

Numerous drugs target EGFR. In particular, two monoclonal antibodies, cetuximab and panitumumab, target the ECD of EGFR. Both of these drugs are FDA-approved for the treatment of metastatic colon cancer, and cetuximab is also FDA-approved for head and neck cancer. In addition, a recent lung cancer clinical trial showed that cetuximab can increase overall survival rate if the tumor shows EGFR overexpression [5], [6]. Many tumors, however, eventually show resistance to cetuximab or panitumumab. Several potential explanations for this phenomenon have been posited: 1) activation of other cell growth pathways [7]; 2) development of mutations in genes involved in the EGFR pathway, including *EGFR* itself [8], [9]; 3) the immune system generating antibodies against the drugs [10]; or 4) the immune system generating antibodies against EGFR ECD, thus masking the drug-binding site [11]. Therefore, there is a need for a blood-based assay that can help evaluate the potential for drug resistance. A study of non-small-cell lung cancer reported that baseline EGFR levels and serum changes in EGFR levels during therapy were associated with response to gefitinib and progression-free survival [12]. However, to our knowledge, the level of association between circulating EGFR and cetuximab or panitumumab, and its significance in treatment response have not been reported. An antibody or aptamer assay that measures free EGFR ECD (i.e., unbound by an EGFR monoclonal antibody) could provide a means to determine how effectively the drug is binding its target.

First discovered more than two decades ago [13], [14], aptamers are nucleic acid molecules with sequence-based unique secondary structures that have a specific binding affinity to targeted proteins. Using the *in vitro* selection method SELEX (Systematic Evolution of Ligands by Exponential Enrichment), highly specific aptamers can be isolated for most proteins. A new generation of aptamers, SOMAmer (Slow Off-rate Modified Aptamers ) reagents, are selected using libraries containing one of four modified dUTPs (benzyl, naphthyl, tryptamino, or isobutyl) that are incorporated into the DNA sequence to provide increased binding affinity, unique secondary structures, high specificity, and decreased dissociation coefficients [15]. SOMAmer reagents can be synthesized in any oligonucleotide synthesizer or ordered from commercial oligonucleotide supply houses.

SOMAmer reagents have several advantages over antibodies currently used for clinical assays. As a synthetic reagent, there is little or no lot-to-lot variability; stability is theoretically nearly infinite; and it is possible to synthesize large quantities of a SOMAmer reagent at a minimal cost [16]. In addition, due to physical similarities between different SOMAmer reagents, it is relatively easy to build multiplex assays.

We describe the first SOMAmer-based clinical assay that detects serum EGFR ECD. An additional characteristic of this assay is that it detects only the drug-unbound fraction of EGFR in sera treated with two widely used EGFR-targeting monoclonal antibody drugs (cetuximab and panitumumab). This feature suggests that the assay could be used as a drug efficacy indicator.

## Materials and Methods

### Ethics Statement

Serum samples for assay development were collected from healthy adults in a Western IRB-approved protocol, and all participants provided written informed consent. This study was exempted from IRB review because we used anonymized samples. Samples tested for both EGFR SOMAmer and ELISA were anonymized samples submitted for EGFR ELISA testing. These samples were presumably from patients with neoplasms. EGFR monoclonal treatment information was retained when available.

### SOMAmer assay

The SOMAmer assay is largely based on the method described previously [16], [17]. The essential steps are to capture the target protein using SOMAmers, wash away unbound proteins and SOMAmers, and release and quantify the protein-bound SOMAmer. SOMAmer reagents are designed with a biotin moiety and a photo-cleavable linker sequence. For this assay, we used a SOMAmer reagent that binds specifically to human EGFR ECD (from SomaLogic, Inc., Boulder, CO). In brief, diluted serum is incubated with SOMAmer affinity reagents, and the bound SOMAmer affinity reagents are captured on a streptavidin-coated plate. Proteins not bound to the SOMAmer affinity reagent (non-EGFR proteins) are washed away, and the captured proteins are labeled with another biotin moiety. The plate is then exposed to LED light to break the link between the biotin and SOMAmer affinity reagent and release both protein-bound and unbound SOMAmer affinity reagents. The supernatant is then transferred to a new streptavidin-coated plate, allowing the now biotinylated protein and streptavidin to interact. The streptavidin-coated plate is then washed to remove protein-unbound SOMAmer affinity reagent and exposed to alkaline buffer to release the protein-bound SOMAmer affinity reagent component, which then serves as the template for quantitative real-time PCR (qPCR).

### qPCR

Unique PCR primers were designed to be complementary within the EGFR SOMAmer sequence. The PCR reactions included *AmpliTaq* DNA polymerase (Life Technologies, Carlsbad, CA) and KOD XL polymerase (EMD Millipore, Billerica, MA) to enhance read-through of modified nucleotides. SYBR® green was purchased from Life Technologies (Carlsbad, CA). qPCR was performed using the ABI ViiA 7 instrument (Life Technologies, Carlsbad, CA).

### EGFR SOMAmer specificity test

#### Equilibrium

Individual equilibration reactions were assembled for each erbB protein/SOMAmer pair, which contained 2 mM AEBSF (4- benzenesulfonyl fluoride hydrochloride, Gold Biotechnology, St. Louis, MO), 5 µM Z-Block (SomaLogic, Inc., Boulder, Co.), 5 µg/mL erbB protein, and 100 nM SOMAmer reagent in 100 µL SB17T. The reaction was incubated at 37°C for 3 hours. After an equilibration reaction, a kinetic challenge was performed where each 100 µL sample was diluted 2× into 10 mM Dextran sulfate for a final volume of 200 µL at 5 mM Dextran sulfate. The sample was mixed and incubated at room temperature for 5 minutes.

#### Protein capture

Following the kinetic challenge, the entire sample volume was transferred to a 96-well filter plate containing SA-agarose beads prewashed in SB17T (please see ref. 15 for recipe). The resulting bead suspension was shaken at 800 rpm for 10 minutes at 25°C in a Fisher ThermoMixer (Thermo Fisher Scientific, Hampton, NH). Using a manually operated vacuum manifold station, the solution was removed by vacuum filtration and then a 200 µL wash (all “washes” performed with 200 µL) was performed with a buffer containing 100 µM biotin in SB17T. After the “biotin wash” was removed by vacuum filtration, three more washes were performed with SB17T, where 200 µL of buffer was added and then removed by vacuum filtration. Two more washes were then performed with SB17T, where the plate was shaken at 800 RPM for 1 minute each before removal of the wash by vacuum filtration. Following the last wash and vacuum filtration, a solution of 0.25 mM NHS-Alexa fluor 647 in SB17T was made freshly and 50 µL was added to each sample. The plate was then shaken at 800 rpm for 10 minutes. During this time the NHS-Alexa fluor 647 was diluted further and added to the purified protein prep for use as a protein standard during gel electrophoresis. After the 10-minute incubation, the solution was removed by vacuum filtration and a “glycine wash” was performed with 10 mM glycine in SB17T. Next a series of 6-SB17T washes was performed; the first four were removed quickly but the last two were incubated with shaking at 800 rpm for 1 minute. After the last wash, 100 µL of SB17T was added to each well; the plate was then exposed to LED light for 5 minutes, rotated 180° for plate uniformity, and exposed for another 5 minutes to photocleave SOMAmer/protein complexes from the SA agarose beads. The photocleaved samples were then spun into a 96-well storage plate by centrifugation.

#### Gel electrophoresis

Polyacrylamide gel electrophoresis was performed on each sample under denaturing conditions. Gels were imaged and quantified using FluorChem Q scanner (Alpha Innotech, San Leandro, CA).

### Drug pre-treatment assay

To assess the effect of drug preincubation on the EGFR SOMAmer assay and the ELISA, prior to the assay serum samples were pre-incubated with cetuximab, panitumumab, or an equal volume of water to final concentrations (in neat samples) of 0.5, 10 and 200 µg/mL. (Note that after the third infusion of drugs during therapy, the mean concentration of both cetuximab and panitumumab in patient plasma at steady state is ∼200 µg/mL.).

### EGFR ELISA

The EGFR ELISA was performed with a commercial kit according to the manufacturer's instructions (Wilex Inc., Cambridge, MA).

### EGFR inter and intra variability measurement

The EGFR interassay variability was calculated by measuring variability from 20 runs of three control serum samples presumed to have low (∼27 ng/mL), middle (∼53 ng/mL), and high (∼310 ng/mL) levels of EGFR. The EGFR intra-assay variability was measured by running low, middle, and high control samples within a single run at least 20 times.

## Results

### Serum EGFR SOMAmer assay

**Figure 1:**
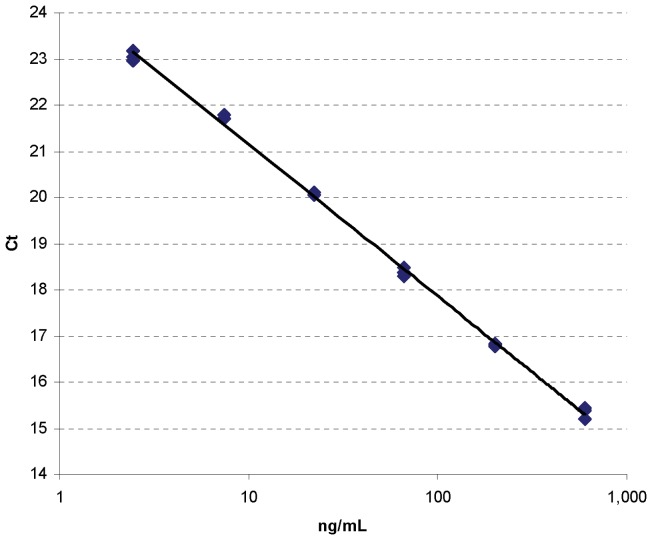
Linear range of the EGFR SOMAmer assay. The dilution series was prepared by diluting purified EGFR in 5-fold steps from 600 to 2.5 ng/mL. Each dilution was further diluted to 30 fold using SB17T buffer to mimic the serum dilution. The EGFR SOMAmer assay and quantitative PCR (qPCR) were done as described in Materials and Methods. Triplicate runs were performed per each dilution.

Our initial goal was to develop a SOMAmer-based quantitative assay that can accurately and specifically quantify serum EGFR ECD levels. Detection and quantitation of EGFR by SOMAmer/qPCR was assessed using serial dilutions of purified EGFR (2.5–600 ng/mL). A typical result is shown in Figure 1. The deviation of the calculated values from the expected values was ≤10%. These data show linearity in the range of 2.5–600 ng/mL.

**Figure 2:**
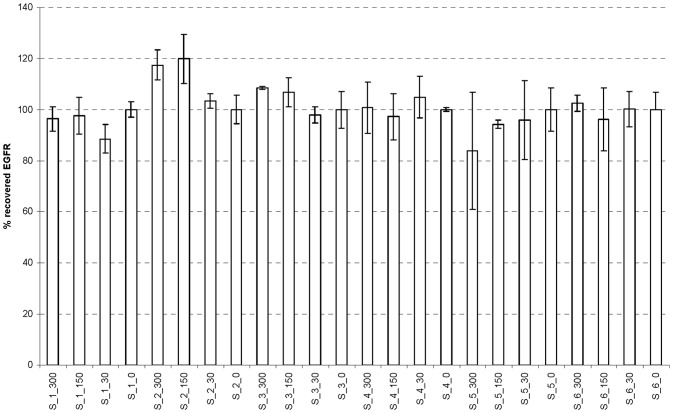
Spiked-in purified EGFR to serum is accurately detected by the EGFR SOMAmer assay. Thirty-fold diluted serum samples were appropriately spiked with three different amounts of purified EGFR ECD (final concentration of 30, 150, and 300 ng/mL plus the endogenous serum EGFR ECD). We then performed the EGFR SOMAmer assay followed by the qPCR. The percent recovery is calculated by dividing SOMAmer-measured EGFR by the expected EGFR level (spiked plus endogenous serum EGFR level), multiplied by one hundred. The codes on the X-axis represent sample number and concentration of spiked EGFR. For example, S_1_300 is serum sample #1 with 300 ng/mL of spiked purified EGFR protein. Each sample was measured three times. The error bars represent standard deviations.

To test the accuracy of the assay, 6 sera from healthy individuals were spiked with 3 different levels of purified EGFR (30, 150, and 300 ng/mL final concentration in neat serum). Expected recovery for each sample was calculated by dividing measured EGFR by the sample's endogenous serum EGFR level plus the spiked-in level. The spiked samples showed recovery of 100±20 %, with a mean recovery of 100.8% (Figure 2).

**Figure 3:**
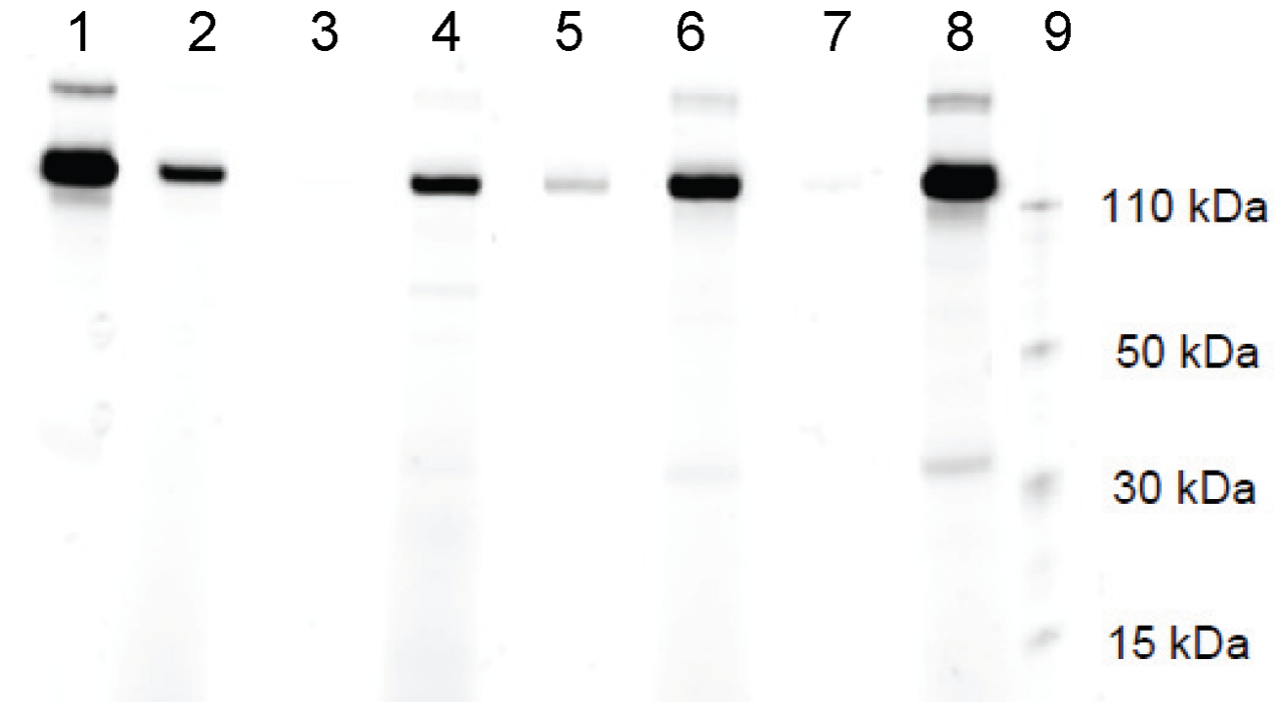
EGFR SOMAmer specificity test. EGFR SOMAmer reagent was incubated with one of the ErbB proteins followed by labeling of bound protein with Alexa fluor 647. After extensive washing, photocleavage dissociates SOMAmer:protein complex from the bound beads, and the complex is separated by polyacrylamide gel electrophoresis under denaturing conditions. Lane 1, pulled down EGFR; Lane 2, EGFR standard; lane 3, pulled down ErbB2; lane 4, ErbB2 standard; lane 5, pulled down ErbB3; lane 6, ErbB3 standard; lane 7, pulled down ErbB4; lane 8, ErbB4 standard, and lane 9, MW size standards The EGFR SOMAmer showed limited cross-reactivity with ErbB family proteins. Relative to EGFR, the SOMAmer “pulled down” limited amounts of ErbB2 (0.4%), ErbB3 (6.9%), and ErbB4 (1.3%).

To test the specificity of the EGFR SOMAmer reagent, we measured its binding affinity for other ErbB family members (ErbB2, ErbB 3, and ErbB 4). The results of polyacrylamide gel electrophoresis under denaturing conditions (Figure 3) demonstrated that the EGFR SOMAmer pulled down much more EGFR (ErbB1, lane 1) than ErbB family members (lanes 3–7). Relative to EGFR, the EGFR SOMAmer pulled down limited amounts of ErbB2 (0.4%), ErbB 3 (6.9%), and ErbB 4 (1.3%). These data suggest that the EGFR SOMAmer can specifically recognize EGFR.

To evaluate variability of the EGFR SOMAmer assay, we measured within-run (intra) and between-run (inter) variability using serum samples containing three levels of EGFR (Low, Middle, and High) (Table 1). In the intra-assay variability study, the % coefficients of variation (CVs) for Low, Middle, and High samples were 11.4, 12.2, and 7.1, respectively (average  = 10.2). In the inter-assay variability study, the % CVs for Low, Middle, and High samples were 17.9, 17.2, and 14.5, respectively (average  = 16.5).

**Table 1:** Intra and inter assay variability of EGFR ECD SOMAmer assay.

		Low	Middle	High
Intra	Average (ng/mL)	26.6	50.9	316.0
	STD	3.0	6.2	22.3
	%CV	11.4	12.2	7.1
Inter	Average (ng/mL)	27.2	52.6	310.2
	STD	4.9	9.1	44.9
	%CV	17.9	17.2	14.5

Inter-assay variability was determined from 20 runs each of control samples with low (∼27 ng/mL), middle (∼53 ng/mL), or high (∼310 ng/mL) concentrations of EGFR. Intra-assay variability was determined by testing each control sample at least 20 times in a single run.

### EGFR SOMAmer assay detects cetuximab and panitumumab-unbound fraction of EGFR

**Figure 4:**
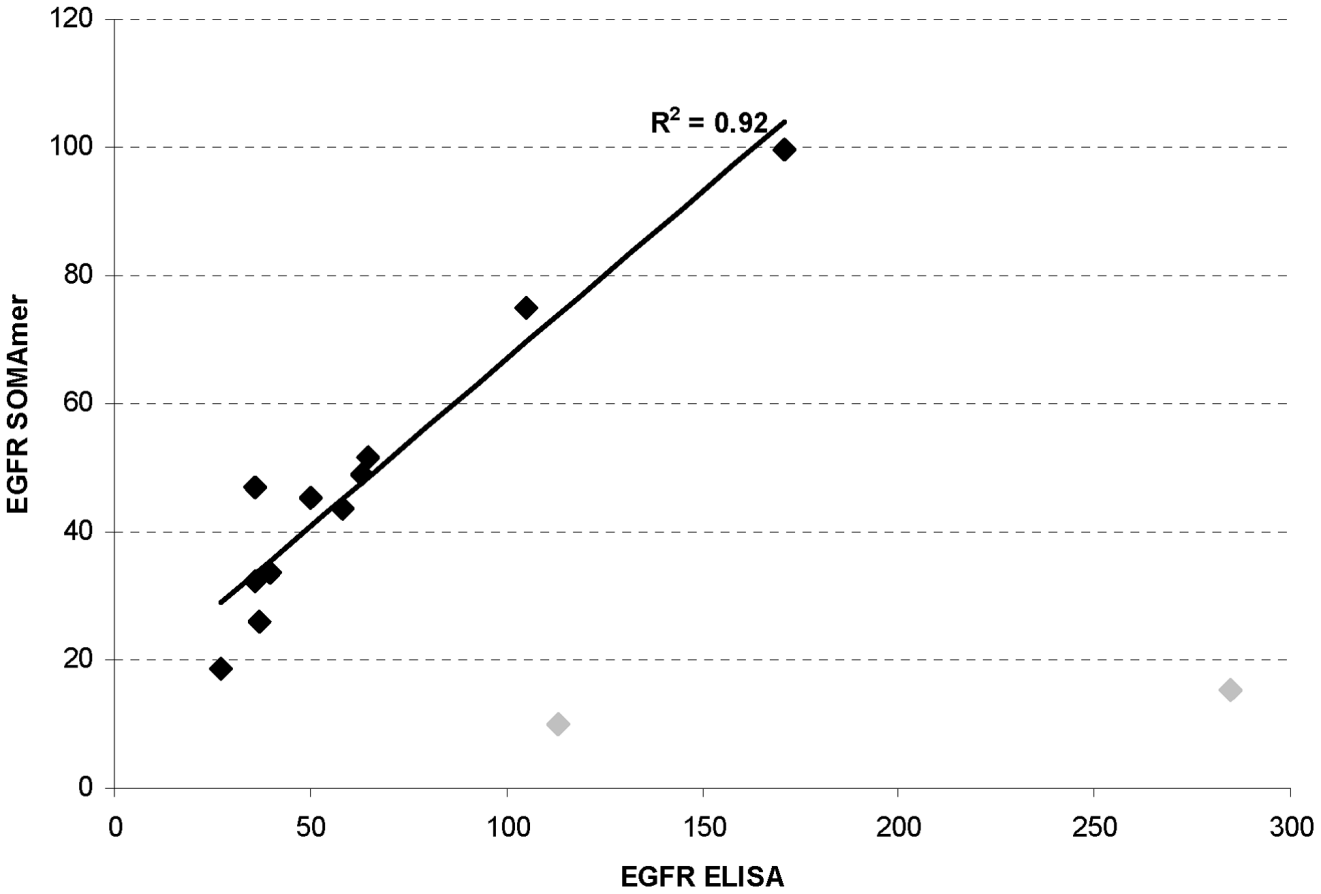
Comparison of ELISA and SOMAmer EGFR levels. Serum EGFR ECD levels were measured using EGFR SOMAmer assay (y-axis) and ELISA (x-axis). Black diamond data points showed correlation between the two methods and were used for R^2^ calculation. Grey diamond data points showed much reduced EGFR SOMAmer levels and were not used for R^2^ calculation.

During the initial assay development, we compared the serum EGFR results between ELISA and the SOMAmer assay using anonymized samples submitted for EGFR analysis. As shown in Figure 4, for most samples we observed good correlation between the two methods (R^2^ = 0.92). However, the ELISA still yielded absolute EGFR levels approximately 10/ng/mL higher than those obtained from the SOMAmer assay. We noted that 2 patient samples showed marked discordance between the two methods (Table 2, samples 1 and 2). In both cases, the SOMAmer assay yielded much reduced levels of EGFR compared to the ELISA assay. Interestingly, the clinical information on these patients showed that they were receiving an anti-EGFR monoclonal drug treatment. Note that not all samples with monoclonal drug treatment showed reduced SOMAmer EGFR levels. For example patient 3 in Table 2 was receiving panitumumab treatment but showed similar EGFR levels with both methods.

**Table 2:** Detection of drug-unbound EGFR in patents receiving treatment with an EGFR monoclonal antibody.

Patient	EGFR, ng/mL		Drug
	ELISA (Total EGFR)	SOMAmer Assay (Unbound EGFR)	
1	285	15	Panitumumab
2	113	6	Cetuximab
3	105	75	Panitumumab

**Figure 5:**
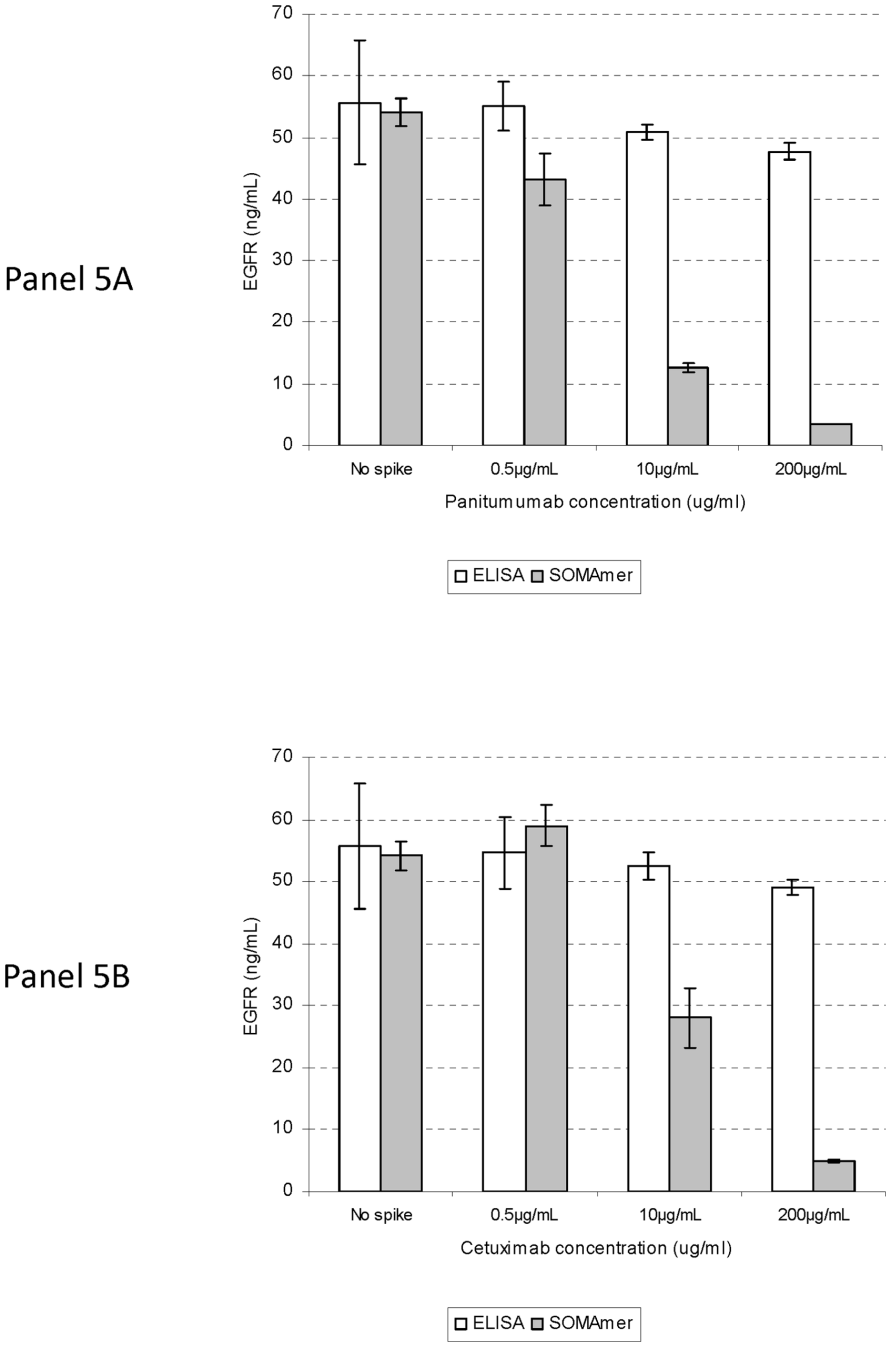
EGFR SOMamer assay detects cetuximab and panitumumab-unbound fraction of EGFR. Normal serum samples were incubated with varying amounts of panitumumab [PANEL A] or cetuximab [PANEL B] for 30 min at room temperature. These samples were then diluted 30 fold, followed by EGFR SOMAmer capture and quantitative PCR (qPCR) (gray bars). EGFR ELISA was also performed on drug-treated samples on the same day (white bars). Triplicate samples were tested at each condition. The vertical lines at each bar represent standard deviations among replicates.

We hypothesized that the SOMAmer assay was measuring primarily drug-unbound EGFR, while the ELISA was measuring both drug-bound and unbound EGFR. To test this hypothesis, we performed the EGFR SOMAmer assay on pooled normal serum after incubation with cetuximab or panitumumab at concentrations ranging from 0.5 to 200 μg/mL (note that the steady-state level of these drugs in patient plasma is ∼200 µg/mL). Both drugs decreased levels of EGFR-captured SOMAmer in a dose-dependent manner (Figure 5A and 5B, gray bars). At 200 µg/mL, both drugs decreased EGFR recovery to less than 10% of baseline levels. At any given drug concentration, EFGR recovery was lower with panitumumab than with cetuximab pretreatment. This finding is consistent with the lower dissociation constant (K_d_) of panitumumab relative to cetuximab [18].

The inverse relationship between the level of drug and EGFR recovery by the SOMAmer assay suggests that both cetuximab and panitumumab interfere with the assay. This interference likely results from masking of the EGFR SOMAmer binding site on EGFR. Therefore the EGFR that is detected in the presence of these drugs likely represents the drug-unbound fraction. We also performed EGFR ELISA on the same samples that had been pre-treated with either cetuximab or panitumumab (Figure 5A and 5B, white bars). Although the recovery of EGFR decreased at higher drug doses, it was a much more moderate decrease: recovery remained above 80% at 200 ng/mL of either drug, suggesting that the EGFR ELISA has much less interference from these EGFR monoclonal antibodies. Overall, our data suggest that, unlike the ELISA, the SOMAmer assay measures primarily cetuximab and panitumumab-unbound EGFR in serum. It is also interesting to note that a substantial level of drug-unbound EGFR can be detected even in patients receiving these drugs (e.g., patient #3 in Table 2).

## Discussion

Aptamers were first discovered more than two decades ago [13], [14]. In therapeutics, an RNA aptamer targeting vascular endothelial growth factor (VEGF) has been approved by the FDA for age-related macular degeneration treatment, and about a dozen aptamers are in late stage clinical trials [19], [20]. In the diagnostics field, aptamer-based assays have not yet entered the clinical arena. In this work, we present, to our knowledge, the first clinical assay that uses a modified aptamer (SOMAmer reagent) as a protein-capturing reagent. SOMAmers have several theoretical and practical advantages over antibodies. First, the fact that they are synthetic reagents and not biologicals leads to decreased costs, decreased lot to lot variability, and infinite reagent supply.

In addition to quantitatively detecting circulating EGFR, the assay described in this article has a potential added benefit in that it detects primarily EGFR that is not bound by cetuximab and panitumumab-unbound. Our data in Figure 5 showing a drug-dose-dependent decrease in the level of detectable EGFR supports such a claim.

We successfully validated the EGFR SOMAmer assay using standard laboratory validation procedures. This assay is highly accurate, as evidenced by the nearly 100% recovery of spiked EGFR from normal sera. Moreover, the minimal interaction of the EGFR SOMAmer with other ErbB family members showed that it is highly specific. The linear detection range was 2.5–600 ng/mL.

Cetuximab and panitumumab are FDA-approved as stand-alone treatments or in conjunction with other therapies for metastatic colorectal cancer; cetuximab is also approved for treatment of metastatic head and neck cancer. However, mutations affecting downstream elements of the EGFR signaling pathway may cause drug resistance. In colorectal cancer, for example, mutations in *KRAS* can cause resistance to both drugs [21]. Although *KRAS* mutation screening is the only assay recommended by the FDA to evaluate treatment eligibility, mutations in other genes such as *BRAF*, *PIK3CA*, and *PTEN* can also lead to drug unresponsiveness [8].

Although most patients who respond to cetuximab and panitumumab eventually acquire drug resistance, acquired mutations affecting components downstream of EGFR in the signaling pathways may explain only part of the situation. Another scenario that could account for drug resistance is suboptimal drug:target interaction. This could happen if the drug or target is masked – for example, by antibodies that target either the drug or the EGFR ECD [10], [22], [23]. Mutations that affect the EGFR ligand-binding site could have a similar effect. A recent study showed that colon cancer patients who underwent cetuximab therapy could acquire a new mutation in the ECD-domain of the *EGFR* gene that blocks binding of EGFR to cetuximab [9]. This mutation has not been detected in cetuximab-naïve patients, suggesting that treatment selects for it. Interestingly, patients who developed such mutations still responded to panitumumab, suggesting that understanding drug:target interactions could change patient management.

Currently, the measurement of free EGFR ECD using SOMAmer technology is available for clinical use. This may provide additional information relative to that provided by ELISA-based measurements in routine clinical use. Eventually measurement of free-EGFR could be useful for evaluating potential drug resistance: the finding of significant levels of unbound EGFR could suggest the need for altering dosage or offering alternative treatments. To explore this possibility, we are currently planning a clinical study to examine the correlation of drug-unbound EGFR with treatment response. This assay could also be useful for routine monitoring of circulating EGFR in cancer patients who are not taking cetuximab or panitumumab.
